# Doxycycline Therapy for Abdominal Aortic Aneurysm: Inhibitory Effect on Matrix Metalloproteinases

**DOI:** 10.7759/cureus.14966

**Published:** 2021-05-11

**Authors:** Smit Paghdar, Taheseen M Khan, Nishant P Patel, Savitri Chandrasekaran, Joaquim Francisco Maria De Sousa, Nicholas Tsouklidis

**Affiliations:** 1 Medicine, California Institute of Behavioral Neurosciences & Psychology, Fairfield, USA; 2 Internal Medicine, Surat Municipal Institute of Medical Education and Research (SMIMER), Surat, IND; 3 Internal Medicine, Government Medical College, Surat, Surat, IND; 4 Internal Medicine, California Institute of Behavioral Neurosciences & Psychology, Fairfield, USA; 5 Surgery, S.S. Institute of Medical Sciences and Research Centre, Davangere, IND; 6 Emergency Medicine, Healthway Hospital, Panaji, IND; 7 Health Care Administration, University of Cincinnati Health, Cincinnati, USA; 8 Medicine, Atlantic University School of Medicine, Gros Islet, LCA

**Keywords:** doxycycline, abdominal, aortic aneurysm, matrix metalloproteinase

## Abstract

Abdominal aortic aneurysm (AAA) is a life-threatening condition associated with smoking, aging, atherosclerosis, and destruction of the connective tissue in the abdominal aortic wall. Disturbances in the synthesis and degradation of matrix metalloproteinase (MMP) have been known to contribute to the development of AAAs. The only available treatment of AAA is surgical repair. Doxycycline, a tetracycline analog, is thought to have an inhibitory effect on MMPs. Knowing the effect of doxycycline, there may be some favorable effects of the drug to reduce the growth of small AAAs and avoid the need for invasive treatment. This article aims to determine the relationship between doxycycline and the MMPs to prevent the growth of small AAAs. We conducted our review using online resources such as PubMed, Google Scholar, The Journal of Vascular Surgery, and ResearchGate. The result of our study supports the effect of doxycycline in preventing the growth of small AAAs. We conclude that therapeutic treatment with doxycycline in patients with small AAAs can prevent the growth of aneurysms, life-threatening aneurysm rupture, and reduce the need for expensive, invasive treatment.

## Introduction and background

**Figure 1:**
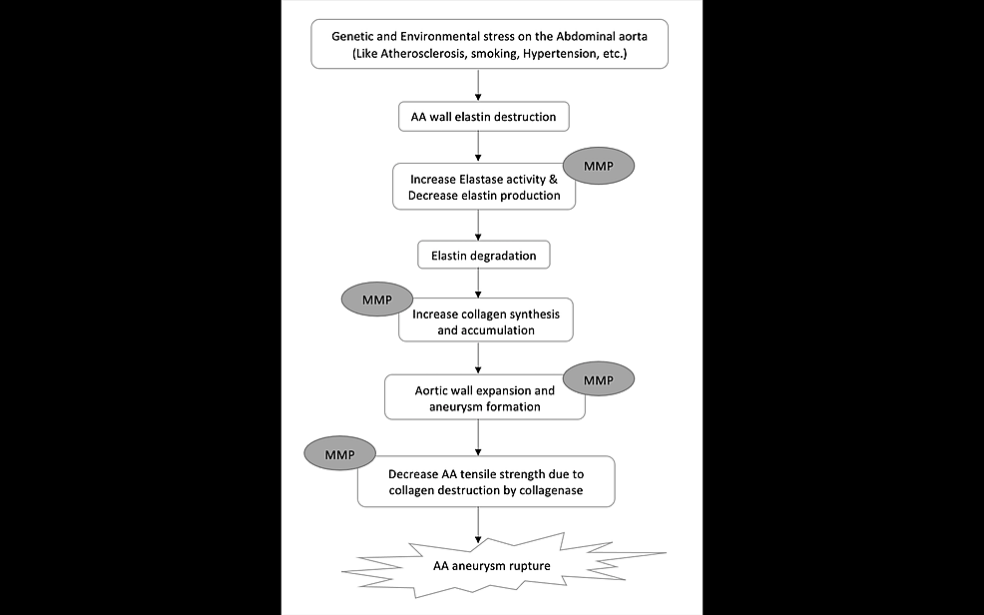
Representation of the pathogenesis of AAA through proteolytic degradation with MMPs. AAA: abdominal aortic aneurysm; AA: abdomial aorta; MMP: matrix metalloproteinase

Abdominal aortic aneurysm (AAA) is a common and serious medical condition in the elderly, especially among males [1]. Aneurysms are usually asymptomatic until they rupture. Rupture is often life-threatening, with mortality ranging from 85% to 90% [2]. AAA is characterized by localized chronic inflammation of the aortic wall [3-6]. They often grow slowly and are characterized by localized dilatation of the abdominal aorta in which the aortic diameter is ≥3.0 cm (normal diameter <3 cm). The pathogenesis of AAA includes inflammation and immune response, smooth muscle cell apoptosis, extracellular matrix degradation, biomechanical wall sheer stress, and molecular genetics. Figure 1 shows the pathogenesis of AAA through proteolytic degradation with matrix metalloproteinases (MMPs).

The diagnosis of AAA is mostly incidental when a physician scans or examines the abdomen for another reason. Surgical repair is currently the only available treatment option for AAA. A more proactive and non-invasive strategy would be to identify AAAs by screening starting at age 65, and then to intervene therapeutically to reduce AAA expansion with preventive measures [7]. Small (diameter of <5.5 cm), asymptomatic AAAs are typically managed conservatively by regular monitoring. Current guidelines recommend surgical repair when the aneurysm diameter expands by more than 1 cm per year or a large diameter of more than 5.5 cm. Nevertheless, surgical treatment may remain the preferred choice of management for patients at a high risk of mortality.

Although elective surgical repair is the only practical approach to prevent deaths from ruptured AAAs, there are no alternative therapeutic strategies for this disease [8]. Because aneurysms are the result of destructive remodeling of the elastic media and outer aortic wall, recent investigations have emphasized disease mechanisms involving chronic aortic wall inflammation and extracellular matrix protein degradation [9,10].

Elastin is a major structural component of the aorta and one of the most substantial proteins of the extracellular matrix [11]. Specific proteinases dissolve elastic fibers, and MMPs are thought to contribute to aneurysm development, including MMP-2, MMP-9, and MMP-12 [12-15]. MMP-9 is the most abundant proteinase secreted by human AAA tissue explants in vitro. It is actively expressed by macrophages located at the site of tissue damage in situ [15]. MMP-9 correlates with increasing aneurysm diameter [16], and its level is elevated in the circulating plasma of patients with AAAs [17]. MMP-12 is also considered important in aneurysm development because it is selectively produced by macrophages within AAA tissue. Unlike other MMPs expressed in the degenerating aortic wall, it is specifically localized to elastin fiber fragments by immunohistochemistry [15]. These observations have speculated that either MMP-9 or MMP-12 might be necessary for aortic wall degeneration and the development of an aneurysm, thereby providing targets for non-invasive pharmacologic therapy [8,10].

The concept of pharmacologic treatment is to prevent the growth of AAA or even reducing aneurysm size and avoiding rupture of an aneurysm. Some studies have indicated that tetracycline derivatives have the ability to stop the progressive degradation of elastin by inhibiting MMPs in the aortic wall, and they do so at clinically approved dose schedules. These study results support the view that MMPs are the targets for pharmacological treatment, and especially tetracyclines might be useful in preventing the growth of AAAs. Hence, this article explores the effectiveness of doxycycline in preventing the growth of small AAAs through several clinical trials and review articles.

## Review

Methodology

PubMed, Google Scholar, Journal of Vascular Surgery, and ResearchGate online databases were used for the purposes of collecting corresponding data. A total of 48 scientific studies resulted from our search of keywords. Keywords included “Doxycycline, abdominal, aortic aneurysm, matrix metalloproteinase.” Of the 48 studies, five were included in our final review article with specific inclusion criteria such as study period between 2000 and 2020, clinical trials, and systemic reviews. All of the included studies met the quality specification and were peer-reviewed.

Results

A total of five studies were selected to evaluate the effect of doxycycline on the growth of AAAs. One study differed from the rest of the studies and reported no effect of doxycycline on the growth of AAAs, while other studies reported a positive effect of doxycycline to prevent the growth of small AAAs. Doxycycline can inhibit selective MMP inhibition, which arrests the growth of small AAAs. Table 1 lists the studies used in this review article, which includes conclusions drawn from the respective studies.

**Table 1:** Description of the studies that met the inclusion and exclusion criteria for this review. AAA: abdominal aortic aneurysm; dox: doxycycline; MMP: matrix metalloproteinase; SMCs: smooth muscle cells

Study	Location	Study period	Sample	Conclusion
Meijer et al. [18]	Netherlands	2013	286 (Patients with AAA diameter 3.5 to 5 cm)	Dox therapy did not reduce aneurysm growth and did not change the need for AAA repair surgery
Abdul-Hussien et al. [19]	Netherlands	2008	60 (Patients with scheduled elective, open AAA repair without chronic inflammatory disease or inflammatory aortic aneurysm)	Short-term dox therapy improves the balance of proteolytic enzymes through an effect on neutrophil content on the aortic wall
Mosorin et al. [20]	Finland	2001	32 (Patients with AAA diameter <5.5 cm)	Favorable effect of dox in the prevention of growth of small AAAs
Curci et al. [21]	United States of America	2000	15 (Patients with scheduled elective AAA repair)	Dox influences the connective tissue degradation within human aneurysm tissue and reduces the growth of small AAAs
Liu et al. [22]	United States of America	2003	Human SMC culture	Dox inhibits MMP-2 expression from cultured human aortic SMCs

Discussion

Many studies have been conducted worldwide to understand the anti-inflammatory effect of doxycycline in preventing AAA growth as it is believed that the pathology of AAA is best described as a continuous, localized inflammatory response that is accompanied by excess MMP activity [23]. The purpose of doxycycline therapy is the notion that MMP-9 is actively and critically involved in AAA formation. This notion is supported by an abundance of MMP-9 in growing AAAs, and the observation of the MMP-9 gene arrests AAA formation in animal models of the disease. Animal studies have shown a beneficial effect of doxycycline preventing AAA growth [24]. However, human studies, including clinical trials and cohort studies, provide conflicting evidence.

Doxycycline and Expression of Matrix Metalloproteinase-9

Studies have found that doxycycline-mediated suppression of AAAs resulted from MMP-9 inhibition [25-29]. A randomized, double-blind, placebo-controlled trial was conducted in 2013 by Meijer et al. [18]. A total of 286 patients with small AAA (aneurysm diameter ranging between 3.5 and 5 cm) were randomly assigned and included in the study: 144 in the doxycycline group (daily dose of 100 mg of doxycycline) and 142 in the placebo group. This study aimed to test whether doxycycline inhibits AAA progression in humans. AAA diameter measurements were taken by ultrasonography at the start of the study and at six-, twelve-, and eighteen-month follow-up visits. This study concluded that long-term doxycycline treatment did not prevent aneurysm progression. This unexpected study finding challenges the validity of the existing models of the human AAA. It differs from other studies that support the use of doxycycline to prevent the growth of small AAAs.

Another clinical trial by Abdul-Hussien et al. aimed to study the effect of doxycycline in improving proteolytic balance by reducing neutrophil content in the abdominal aortic wall [19]. They randomly assigned 60 patients undergoing elective AAA repair into four groups of low- (50 mg/dL), medium- (100 mg/dL), or high-dose (300 mg/dL) doxycycline versus no medication. The study reported that after two weeks of doxycycline treatment before the aneurysm repair surgery reduces aortic wall MMPs and selectively suppresses neutrophil collagenase and gelatinase (MMP-8 and MMP-9) protein levels. It also increases protein levels of the protease inhibitors tissue inhibitor of MMP-1 and cystatin C. This is confirmed by immunohistochemical analysis of aortic wall aneurysm tissue samples obtained during the surgery, which revealed a 75% reduction in aneurysm wall neutrophil content than the samples obtained from the placebo group patients. The study concluded that short-term preoperative doxycycline therapy helps reduce the growth of small AAAs, presumably through an effect on aortic wall neutrophil content. Hence, it provides a rationale for doxycycline treatment in patients with small AAAs.

In addition to inhibition of MMPs, eradication of *Chlamydia pneumoniae* infection with doxycycline has the advantage of preventing the growth of small AAAs. *C. pneumoniae* infection may be responsible as an initiating or aggravating factor in the process of AAA formation and expansion [30]. A prospective, double-blind, randomized, placebo-controlled study was conducted by Mosorin et al. [20]. The objective of the study was to investigate the efficacy of doxycycline in reducing the expansion of small AAAs. A total of 32 eligible patients were randomly assigned to receive either doxycycline (150 mg daily) or placebo during a three-month period and examined during an 18-month period by performing an ultrasound. Outcome measures were grouped into aneurysm expansion rate, the number of patients who had AAA rupture or repair, *C. pneumoniae* antibody titers, and serum concentration of C-reactive protein (CRP). The results of this study suggest that the overall aneurysm expansion rates during the last two six-month periods were significantly lower in the doxycycline group than in the placebo group. Results based on the *C. pneumoniae* antibody titer reveal that elevated IgG titers against *C. pneumoniae* were significantly associated with increased expansion rate in the placebo group. According to the serum concentration of CRP, results suggest that CRP values three months after the doxycycline treatment showed a significant decrease from the baseline values. In contrast, CRP values increased in the placebo group. This study concluded that doxycycline may favorably prevent the growth of the small AAAs and can be useful to avoid the need for surgical treatment. However, the small size of the study cannot justify the use of doxycycline treatment to reduce small AAAs.

Another study was performed by Curci et al. to determine doxycycline suppression of MMPs within human aneurysm tissue and explain the molecular mechanisms underlying this effect [21]. Aneurysm tissues were collected from 15 patients with an AAA. Eight were treated with doxycycline before surgery (100 mg orally twice for seven days) and seven were treated with a placebo. MMP activity was examined using gelatin zymography and immunoblot analysis, while RNA was analyzed using reverse transcription-polymerase chain reaction (RT-PCR). This study included examining the effects of doxycycline on the production of MMP in human THP-1 (THP-1 designates a spontaneously immortalized monocyte-like cell line) mononuclear phagocytes in vitro. The results suggest that doxycycline was associated with a slight reduction (24.4%) in the activity of MMP-2 utilizing zymography. At the same time, a 2.5-fold decrease in MMP-9 was reported in doxycycline-treated patients using immunoblot analysis. A 5.5-fold (81.9%) reduction in MMP-9 messenger RNA in doxycycline-treated patients was reported using RT-PCR. They also found that when cultured THP-1 monocytes stimulated with phorbol ester, there was a decrease in MMP-9 protein and mRNA expression after doxycycline exposure in vitro. This study concluded that doxycycline may regulate connective tissue degradation within AAA tissue by controlling the monocyte/macrophage expression of MMP-9 mRNA and suppressing the post-translation activation of proMMP-2. Hence, doxycycline treatment can be useful in preventing the growth of AAAs by MMP inhibition.

Doxycycline and Expression of Matrix Metalloproteinase-2

A study conducted by Liu et al. established the relation between the inhibitory effect of doxycycline on MMP-2 expression from cultured human aortic smooth muscle cells (SMCs) and human aortic aneurysm tissue explant [22]. Human SMC culture was obtained from infrarenal aorta from transplant donors, and monocytes and lymphocytes were separated for the study. Monocytes were cultured for seven days with other co-culture to get a conditioned medium treated with doxycycline at concentrations ranging from 5 to 24 µg/mL for 24 hours. The results show that doxycycline at this concentration directly inhibited MMP-2 production from cultured human aortic SMCs and explanted AAA tissue. They also noted decreased secretion of MMP-9. These findings correlate with the animal study of Petrinec et al. [25]. They found that doxycycline inhibits development in an elastase-induced rat AAA model associated with decreased MMP-2 and MMP-9 production. The study concluded that doxycycline can be useful in clinical trials designed to inhibit the growth of small AAAs and eventually can be offered as a therapeutic treatment for patients with small AAAs to prevent AAA ruptures.

Limitations

In this review article, the studies did not have a large enough sample size to establish the effect of doxycycline in preventing AAA growth. The safety and side effects of long-term doxycycline use is yet to be proved. In addition, the mode of action of the drug is still unclear. The studies only focus on small-sized AAAs. We would need more clinical trials in the near future, which exclude all limitations and provide solid evidence that we can use doxycycline to prevent the growth of AAAs.

## Conclusions

In this review article, our aim was to evaluate the effect of doxycycline on preventing the growth of AAAs. Doxycycline has an anti-inflammatory effect by inhibiting the expression of MMP on the wall of the aortic aneurysm, which is responsible for the growth of AAAs. We found that doxycycline improves the balance between MMP degradation and deposition by selectively inhibiting MMP-9 and MMP-2 expression on the aortic aneurysm wall, which reduces the neutrophil content on the wall, thereby reducing its inflammatory effect and preventing the growth of AAAs. We also find that doxycycline reduces the expression of MMP-9 mRNA, which further supports the anti-inflammatory effect of doxycycline. We did not find the exact mechanism of doxycycline preventing the growth of small AAAs. Hence, we conclude that doxycycline prevents the growth of small AAAs (less than 5.0 cm in size). Thus, we can introduce doxycycline as a therapeutic treatment of small AAAs to prevent the development of a large aneurysm, rupture of a large aneurysm, and prevent the need for invasive treatment of the large aneurysm (more than 5.0 cm in size), which will benefit patients from life-threatening large aneurysmal rupture, as well as socioeconomically by avoiding the invasive surgery. Further research on this subject is required to strongly support the exact mechanism of action and long-term side effects of doxycycline in the treatment of AAAs. A study with a large sample size is required to evaluate the significant positive anti-inflammatory effect of doxycycline to reduce the growth of AAAs.
